# Expanded endoscopic endonasal transsphenoidal approach to determine morphological characteristics and clinical considerations of the cavernous sinus venous spaces

**DOI:** 10.1038/s41598-022-21254-9

**Published:** 2022-10-06

**Authors:** Guowen Zhan, Shanshan Guo, Honglei Hu, Jianchun Liao, Ruishan Dang, Youxiong Yang

**Affiliations:** 1 Department of Otolaryngology-Head and Neck Surgery, Ningbo Yinzhou Second Hospital, 1st North Qianhe Road, Yinzhou District, Ningbo, 315100 Zhejiang People’s Republic of China; 2 Department of Otolaryngology-Head and Neck Surgery, the Affiliated Changzheng Hospital of Second Military Medical University, Shanghai, 200003 People’s Republic of China; 3 Department of Human Anatomy, Second Military Medical University, Shanghai, 200433 People’s Republic of China

**Keywords:** Anatomy, Neurology

## Abstract

The study aimed at investigating the morphological characteristics and interconnected regularities of the cavernous sinus (CS) venous spaces using an expanded endoscopic endonasal transsphenoidal approach. Surgical dissections were performed for 15-colored silicon-injected human head specimens. The CS venous spaces were examined for their morphological and clinical characteristics using an expanded endoscopic endonasal transsphenoidal approach. The intracavernous course of the internal carotid artery (ICA) divided the CS venous spaces into four interconnected virtual compartments: medial, anteroinferior, posterosuperior, and lateral. The CS venous spaces had peculiar morphological characteristics; the medial compartment was C-shaped while the anteroinferior compartment resembled a boat's bow. The mean distances from the medial border of the inferior horizontal segment of cavernous ICA to the mid-line of the pituitary gland (PG) were 6.07 ± 1.61 mm (left) and 5.97 ± 1.89 mm (right); the mean distances from the medial border of the subarachnoid segment of cavernous ICA to the mid-line of the PG were 5.77 ± 1.16 mm (left) and 5.63 ± 1.17 mm (right); the mean distances from the medial border of the anterior vertical segment of cavernous ICA to the mid-line of the PG were 10.27 ± 1.74 mm (left) and 10.47 ± 1.90 mm (right). Morphological characteristics and the knowledge of the interconnected regularities of the CS venous spaces may help surgeons accurately locate the neurovascular structure, and thus may contribute to the effective prediction of tumor invasion and extension during endoscopic CS surgery.

## Introduction

Expanded endoscopic endonasal approaches have become increasingly popular in the last decade as they facilitate access to virtually any region of the ventral skull base including the anterior cranial fossa, parasellar region, clival region, and craniovertebral junction^1,2^. Advances in endoscopic endonasal techniques, image-guided systems, neuronavigation, and customized surgical instruments have enabled surgeons to treat skull base lesions with lesser morbidities^3,4^. The surgeons benefit from the advantages of this approach which include direct access to the ventral skull base by avoiding brain and brainstem retraction, near-field magnification, better surgical field illumination, and minimal manipulation of neurovascular structures^5–7^.

Advances in the expanded endoscopic endonasal transsphenoidal approach have provided for a new route to access the lesions in anatomically complicated structures in the skull base, which are close to the internal carotid arteries, pituitary gland, abducens nerve, and CS. However, because of the depth of the target location and the complexity of the critical regional anatomy, the exposure and resection of these tumors pose a technical challenge to the neurosurgeons and otolaryngologists^8–11^. Space-occupying lesions within this region may distort normal anatomy, and thus stereotactic image guidance can be misleading. Hence, there is a need for more reliable anatomical landmarks for surgery^12–14^. While the different walls of the CS, the intracavernous ICA, and its branches are well described^15–17^, morphological and clinical characteristics of the CS venous spaces, remain relatively unknown.

Our previous study has been investigated the relationship between the morphological characteristics of the sphenoid sinus and endoscopic localization of the cavernous sinus, and we believe that the opticocarotid recess (OCR) and C-shaped ICA in the lateral wall of the sphenoid sinus are the 2 reliable anatomic landmarks in the intraoperative location of the parasellar region of CS^18^. While in this study, using an expanded endoscopic endonasal transsphenoidal approach, surgical dissections were performed on adult cadaver heads to examine the morphological and clinical characteristics of the four compartments of the CS venous spaces. These may have implications for safe surgical procedures with minimal invasiveness.

## Materials and methods

This study was approved by the Ethics Committee of Ningbo Yinzhou Second Hospital (Ningbo, China, 2022003) per the Declaration of Helsinki of 1964 (revised, Hong Kong, 1989). The human cadavers used in the study were supported by the department of Human Anatomy, Second Military Medical University, and informed consents were obtained from all donors or next kin in accordance with relevant guidelines and regulations. 30 specimens from 15 randomly selected adult formalin-processed cadaver heads. Red latex was injected into the internal carotid arteries and blue latex in the internal jugular veins. All anatomical dissections were performed using an expanded endoscopic endonasal transsphenoidal approach in the operating room of the Second Yinzhou Hospital. A rigid endoscope (Karl Storz GmbH and Co., Tuttlingen, Germany), 4 mm in diameter, 18 cm in length, equipped with 0° lenses, was used. A fiberoptic cable connected the endoscope to a light source, and a camera fitted with high-definition second-generation sensors. The video camera was connected to a monitor which displayed a high-definition image. Cadaveric specimens were placed in a supine position; the head was placed at 10 to 15 degrees adducted toward the left shoulder, in a neutral position. Using the bilateral nostril technique, we introduced the endoscope and instruments through the two nostrils. The dissection was performed in four stages as follows: exposure of the anterior, posterior, and lateral walls of the sphenoidal sinus; exposure of the medial and anteroinferior walls of the CS; exposure of the medial and anteroinferior compartments of the CS venous spaces; exposure of the posterosuperior and lateral compartments of the CS venous spaces. A custom-made 1 mm × 1 mm grid was designed on a computer and printed on a transparent acetate sheet. This was used to measure the distances from bilateral cavernous ICAs to the mid-line of the PG. Statistical analyses were carried out using the GraphPad Prism 5 (Version 5.01, GraphPad Software, Inc., USA), and the data were presented as mean ± standard deviation (SD). Analysis for statistical significance was performed using the paired t-test and differences were considered as statistically significant for p < 0.05.

## Results

### Stage 1: Exposure of the anterior, posterior, and lateral walls of sphenoidal sinus

**Figure 1:**
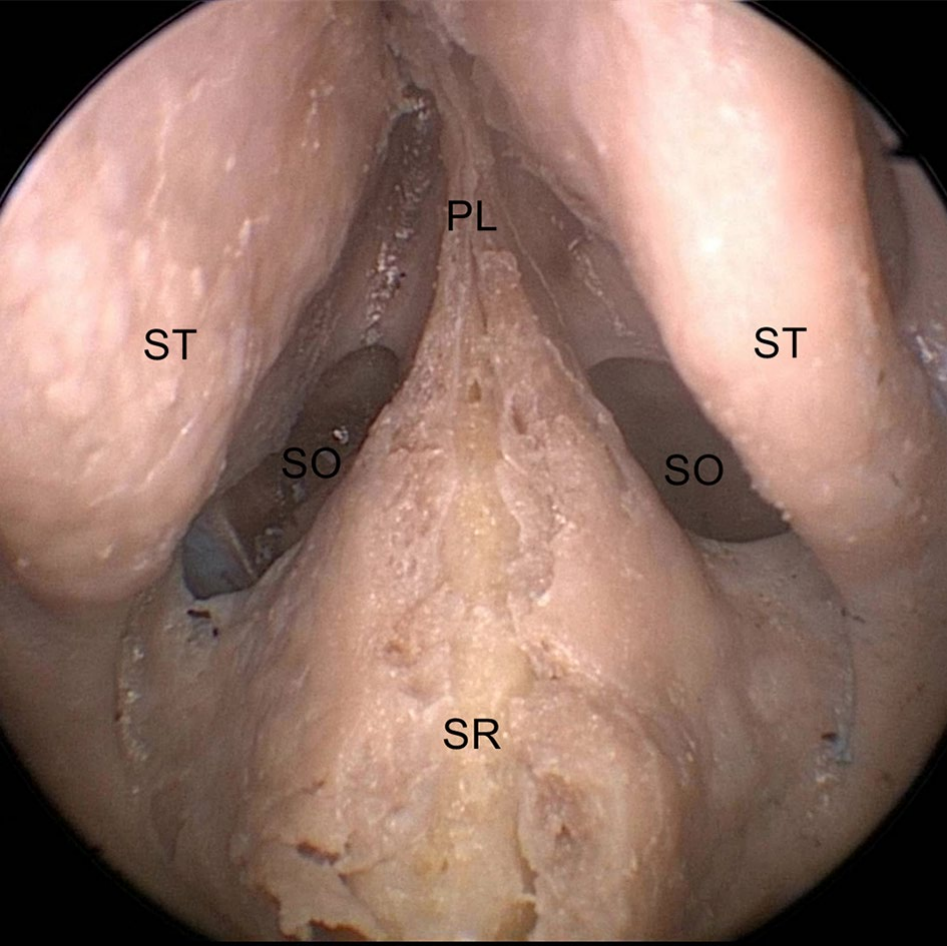
Exposure of the sphenoidal sinus anterior wall in endoscopic view. *ST* superior turbinate, *PL* perpendicularis lamina, *SR* sphenoidal rostrum, *SO* Sphenoid ostia.

The middle and superior turbinates were laterally retracted, and the sphenoid Ostia were bilaterally identified. Septal mucosal cuts were made 20 mm in front of the ostium, 1 mm above the palate, and 2–3 mm below the cribriform plate (until they met anteriorly), for nasoseptal flap harvesting (Fig. 1).

**Figure 2:**
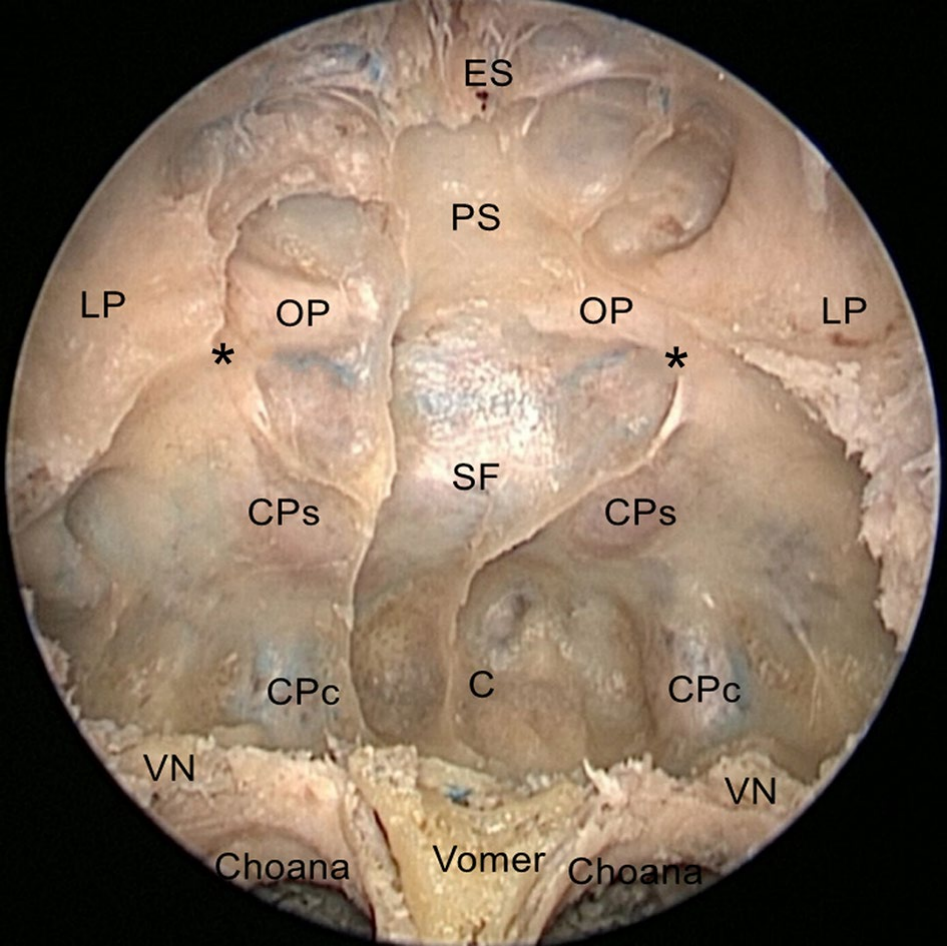
Endoscopic view of the sphenoidal sinus posterior, lateral walls, key anatomical bony landmarks were identified after complete posterior ethmoidectomy, and sphenoidotomy. *PS* Planum sphenoidale, *OP* optic prominence, *CPs* parasellar segment of the carotid prominence, *CPc* paraclival segment of the carotid prominence, *SF* sellar floor, *C* clivus, *LP* lamina papyracea, *ES* ethmoid sinus, *VN* vidian nerve. *Opticocarotid recess.

The sphenoid sinus at the center of the cranial base was surrounded by numerous neurovascular structures. The bony protuberances of the posterior and lateral walls of the sphenoidal sinus were exposed using complete posterior ethmoidectomy, and wide anterior sphenoidotomy. (Fig. 2).

### Stage 2: Exposure of the medial and anteroinferior walls of the CS

**Figure 3:**
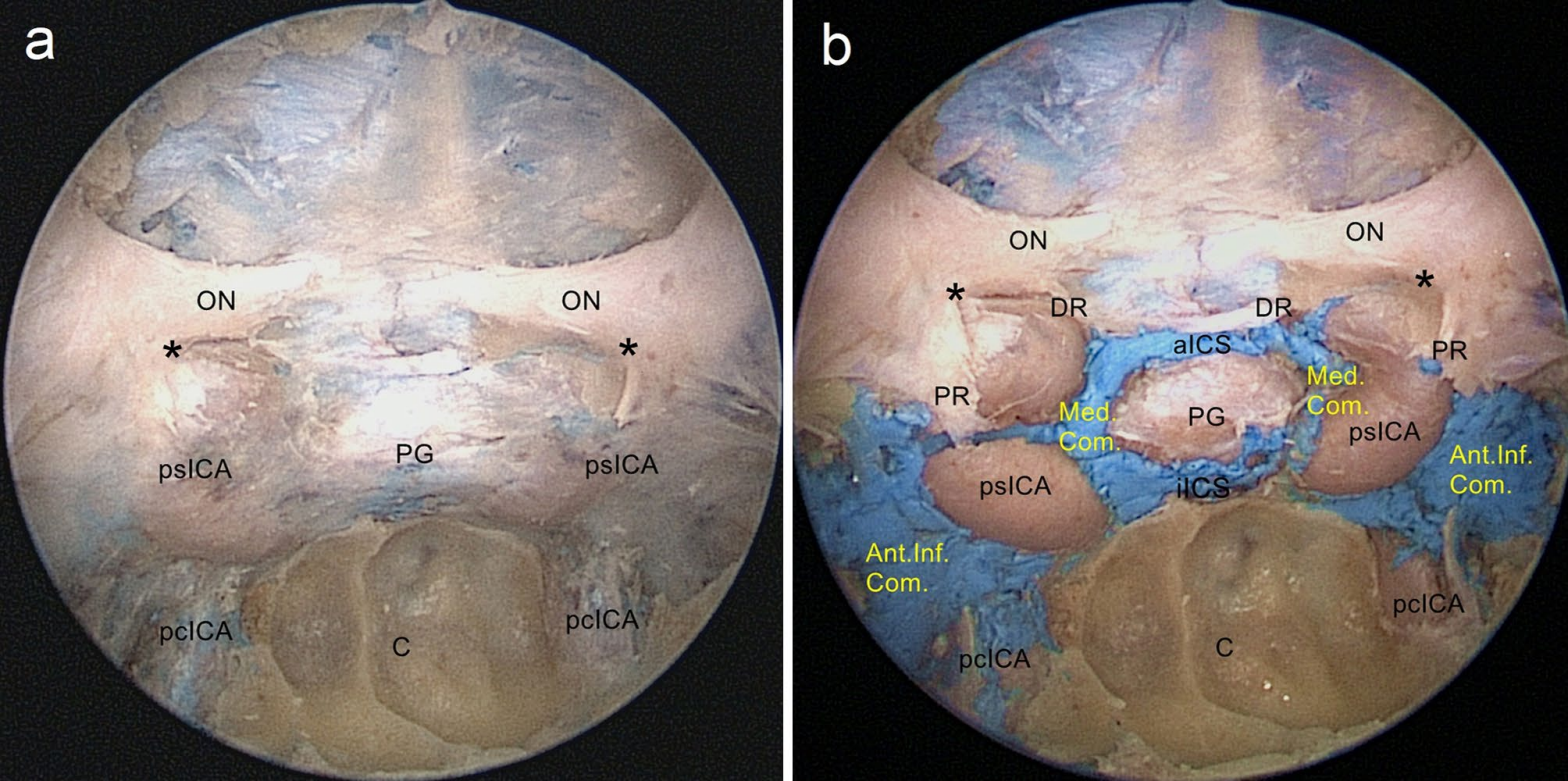
Endoscopic endonasal view of the ICAs, CSs and ICSs. (**a**) After the bone overlying the sphenoidal sinus and sellar floor is removed, the dural layer is shown. (**b**) The layer and latex covering the anterior portion of the pituitary gland and the medial wall of the CS, are resected to expose the CS compartments. ICSs are venous interconnections between the bilateral CSs. *C* clivus, *ON* optic nerve, *psICA* parasellar segment of the ICA, *pcICA* paraclival segment of the ICA, *PG* pituitary gland, *DR* distal ring, *PR* proximal ring, *aICS* anterior intercavernous sinus, *iICS* inferior intercavernous sinus, *Med.Com.* medial compartment of the CS, *Ant.Inf.Com.* anteroinferior compartment of the CS. *Opticocarotid recess.

When the bone overlying the sphenoidal sinus and sellar floor was removed, the dural layer was exposed and it enveloped the anterior surface of the pituitary gland and the medial wall of the CS. The layer in front of the pituitary gland consists of loosely attached two-layered dura; the external layer called the meningeal dural layer is thicker and extends outwards to the medial wall of the CS; the internal layer is transparent, and continuous with the lateral wall of the pituitary gland (Fig. 3a).

The layer and latex covering the anterior portion of the pituitary gland and the medial wall of the CS were resected to expose the bilateral ICAs, CSs, and Intercavernous sinuses (ICSs). The ICAs associated with the sphenoid sinus included the parasellar ICA (psICA), and the paraclival ICA (pcICA); psICA could be subdivided into four segments: the hidden segment, the inferior horizontal segment, the anterior vertical segment, and the superior horizontal segment which consists of the clinoidal segment as well as the intracranial subarachnoid segment; pcICA could be subdivided into two segments: the lacerum and the trigeminal segments^19^. Intercavernous sinuses were the venous interconnections between the bilateral cavernous sinuses in the dura mater around the pituitary gland. (Fig. 3b).

### Stage 3: Exposure of medial and anteroinferior compartments of the CS venous spaces

**Figure 4:**
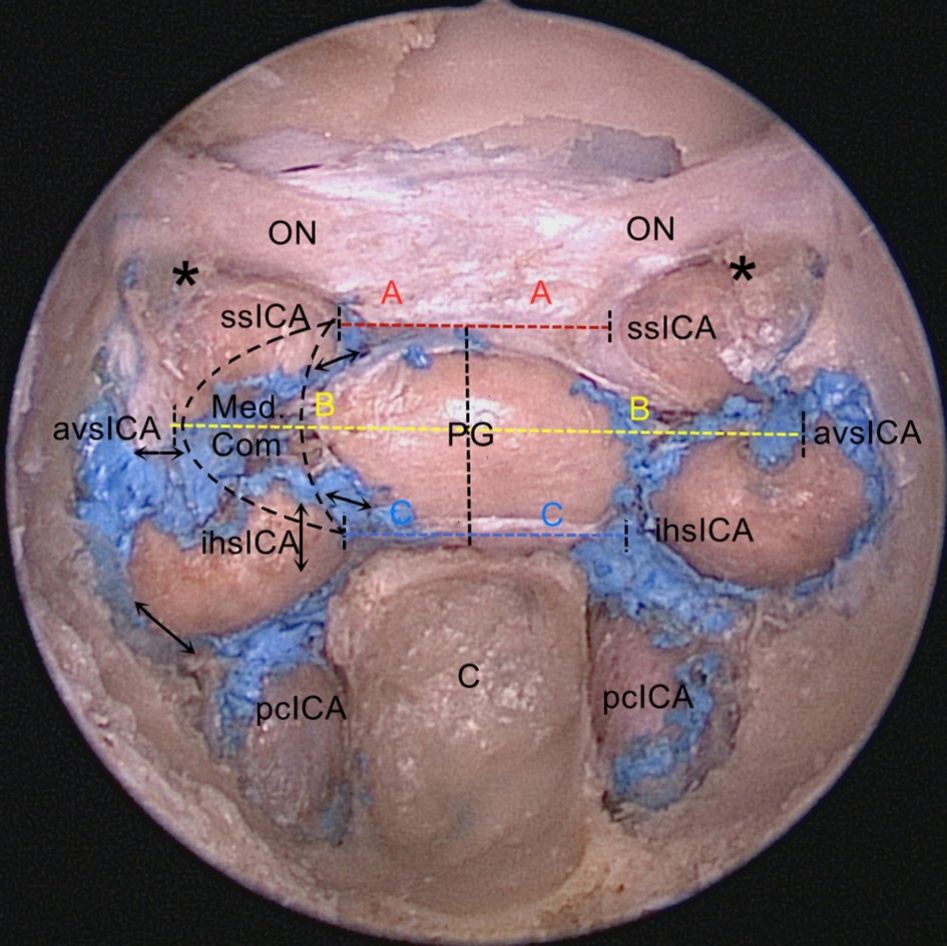
Measurements of the distances between the bilateral cavernous ICAs to the mid-line of the PG in endoscopic view and the medial compartment resembles C-shape (the black dotted line), A (the red dotted line) represents the distance from the medial border of the upper cavernous ICA (subarachnoid segment) to the mid-line of the PG, B (the yellow dotted line) is the distance between the medial border of the anterior vertical segment of cavernous ICA to the mid-line of the PG, C (the blue dotted line) marks the distance from the medial border of the lower cavernous ICA (inferior horizontal segment) to the mid-line of the PG, the medial C-shaped compartment communicates with the anteroinferior compartment anteroinferiorly, the lateral compartment laterally and the posterosuperior compartment posteriorly, also crosses the anterior and inferior intercavernous sinus medially to the opposite compartment (arrows). *PG* pituitary gland, *C* clivus, *ON* optic nerve, *ihsICA* inferior horizontal segment of the ICA, *ssICA* subarachnoid segment of the ICA, *pcICA* paraclival segment of the ICA, *Opticocarotid recess.

**Figure 5:**
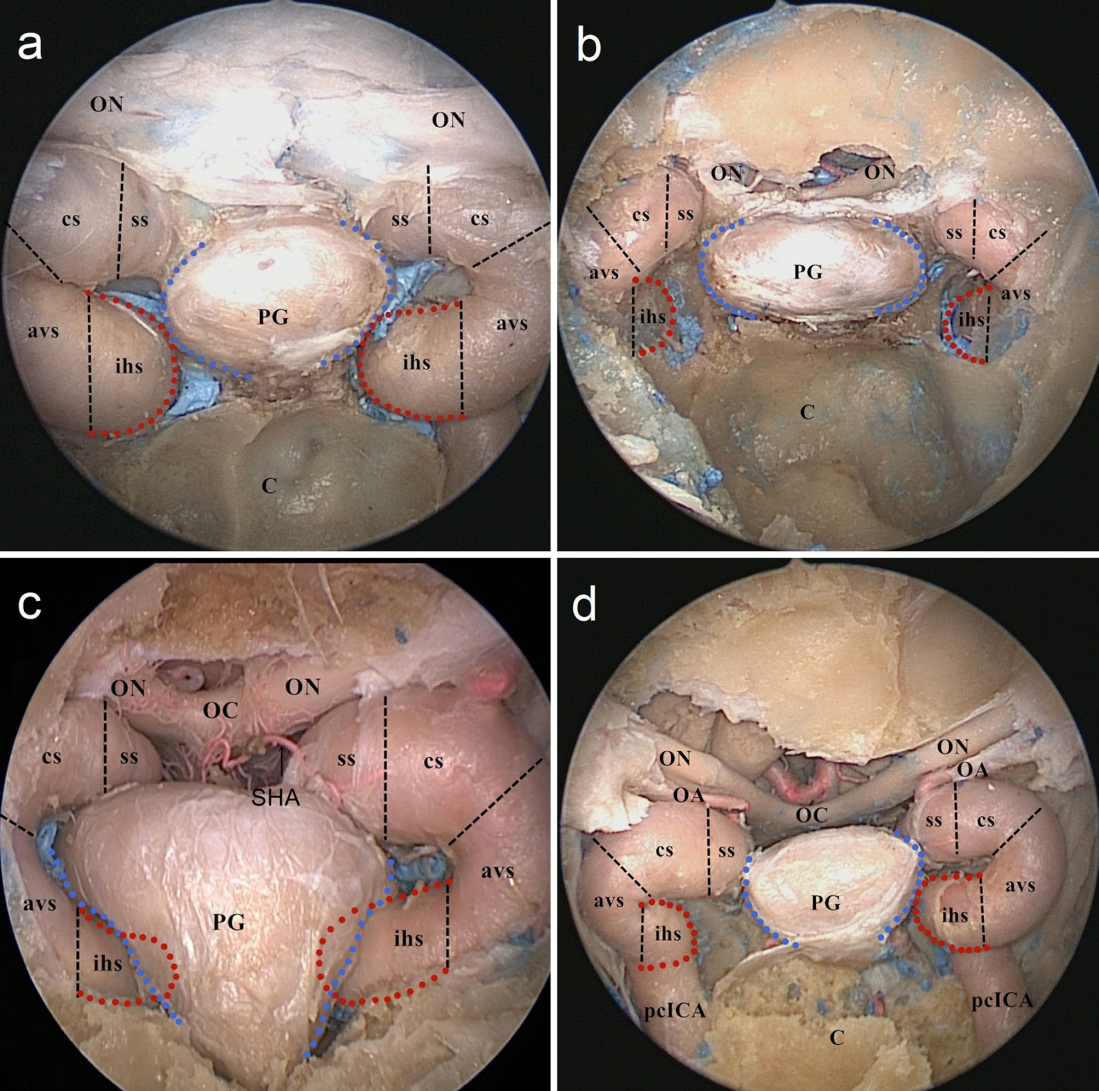
The relationship between the lateral surface of the pituitary gland (PG) and the inferior horizontal segments of the internal carotid artery (ihsICA). (**a**) the bilateral ihsICAs (the red dotted line) are attached contacts with the lateral surface of the PG (the blue dotted line) corresponding to contiguity. (**b**) the bilateral ihsICAs are separated from the lateral surface of the PG corresponding of separation. (**c**) the bilateral ihsICAs are extended to the lateral surface of the PG corresponding to indentation. (**d**) the right ihsICA is separated from the lateral surface of the PG whereas the left one is close contact. *ON* optic nerve, *OC* optic chiasm, *OA* ophthalmic artery, *SHA* superior hypophyseal artery, *PG* pituitary gland, *C* clivus, *pcICA* paraclival segment of the ICA, his inferior horizontal segment of the ICA, *avs* anterior vertical segment of the ICA, *cs* clinoidal segment of the ICA, *ss* subarachnoid segment of the ICA.

Based on their relationship with the cavernous ICA, four CS compartments were classified as follows: medial, anteroinferior, posterosuperior, and lateral compartments. The layers covering the medial and anteroinferior walls of the CS were resected to expose the medial and anteroinferior compartments of the CS venous spaces. The medial compartment of the CS venous space was located between cavernous ICA and pituitary gland, and was superiorly limited by the distal ring, and inferiorly by the inferior horizontal psICA. This compartment lacked nerves, and thus represented a medial surgical corridor owing to a minimal risk of neural damage. Antero–posteriorly, the medial compartment was C-shaped (70%) and non-C-shaped (30%). The medial C-shaped compartment, anteroinferiorly communicates with the anteroinferior compartment, laterally with the lateral compartment, and posteriorly with the posterosuperior compartment; it also medially crosses the anterior and inferior intercavernous sinuses to the opposite compartment (Fig. 4). The superior hypophyseal artery arising from the ICA located above the medial compartment ran in the preinfundibular space and provided the maximum blood supply to the infundibulum, optic chiasm, and proximal optic nerve (Fig. 5c).

A custom-made 1 mm × 1 mm grid was used to measure the distances from the bilateral cavernous ICAs to the mid-line of the PG and the data are presented as mean ± SD. The mean distance A, from the medial border of the lower cavernous ICA (inferior horizontal segment) to the mid-line of the PG was 6.02 ± 1.73 mm, the mean distance C; from the medial border of the upper cavernous ICA (subarachnoid segment) to the mid-line of the PG was 5.70 ± 1.15 mm; the mean distance B; from the medial border of the anterior vertical segment of cavernous ICA to the mid-line of the PG was 10.37 ± 1.79 mm (Fig. 4). There were no significant differences between the left and the right sides. All measurements are displayed in Table 1.

**Table 1:** Measurements of the distances between bilateral cavernous ICAs to the mid-line of the PG in endoscopic view.

Specimen no.	Distance A (mm)		Distance B (mm)		Distance C (mm)	
	Left	Right	Left	Right	Left	Right
1	4	4.5	9	8	4.5	4
2	5.5	6.0	13	13	6.5	6.5
3	5.5	5.0	11.5	11.5	5	4
4	7	5	10	10.5	6.0	6.5
5	6.5	7	7	7	5	4
6	3	3	9	8	3.5	4.0
7	6	6.5	12	13	5.5	5
8	5	6	11.5	11.5	6.5	6.5
9	7.0	6.0	12	13	7.5	8.5
10	6.5	5.5	9	10	5.0	4.5
11	7	8	12	12	10.5	10.5
12	6.5	6.5	8	10	5.5	5
13	5	5	11	9	6.5	7.5
14	6.5	5.5	10	10.5	6.5	6.5
15	5.5	5	9	10	7	6.5
Mean ± SD	5.77 ± 1.16	5.63 ± 1.17	10.27 ± 1.74	10.47 ± 1.90	6.07 ± 1.61	5.97 ± 1.89
Total	5.70 ± 1.15		10.37 ± 1.79		6.02 ± 1.73	

Distance A represents the distance from the medial border of the subarachnoid segment of the ICA to the mid-line of the PG. Distance B is the distance between the medial border of the anterior vertical segment of cavernous ICA to the mid-line of the PG. Distance C marks the distance from the medial border of the inferior horizontal segment of the ICA to the mid-line of the PG.

The relationship between the lateral surface of the pituitary gland (PG) and the inferior horizontal segment of the ICA (ihsICA) was stratified into three types: contiguity (40%, 12/30), separation (26.7%, 8/30), and indentation (33.3%, 10/30). Notably, the relationship between the lateral surface of the PG and bilateral ihsICAs were not always consistent, (20%, 3/15), as shown in Table 2 and Fig. 5.

**Table 2:** The relationship between the lateral surface of the pituitary gland (PG) and the inferior horizontal segments of the internal carotid artery (ihsICA).

Specimen no.	The relationship between the lateral surface of PG and ihsICA	
	Left	Right
1	Contiguity	Contiguity
2	Indentation	Indentation
3	Indentation	Indentation
4	Separation	Separation
5	Separation	Indentation
6	Indentation	Indentation
7	Indentation	Indentation
8	Contiguity	Contiguity
9	Contiguity	Separation
10	Contiguity	Contiguity
11	Separation	Separation
12	Separation	Separation
13	Indentation	Contiguity
14	Contiguity	Contiguity
15	Contiguity	Contiguity

**Figure 6:**
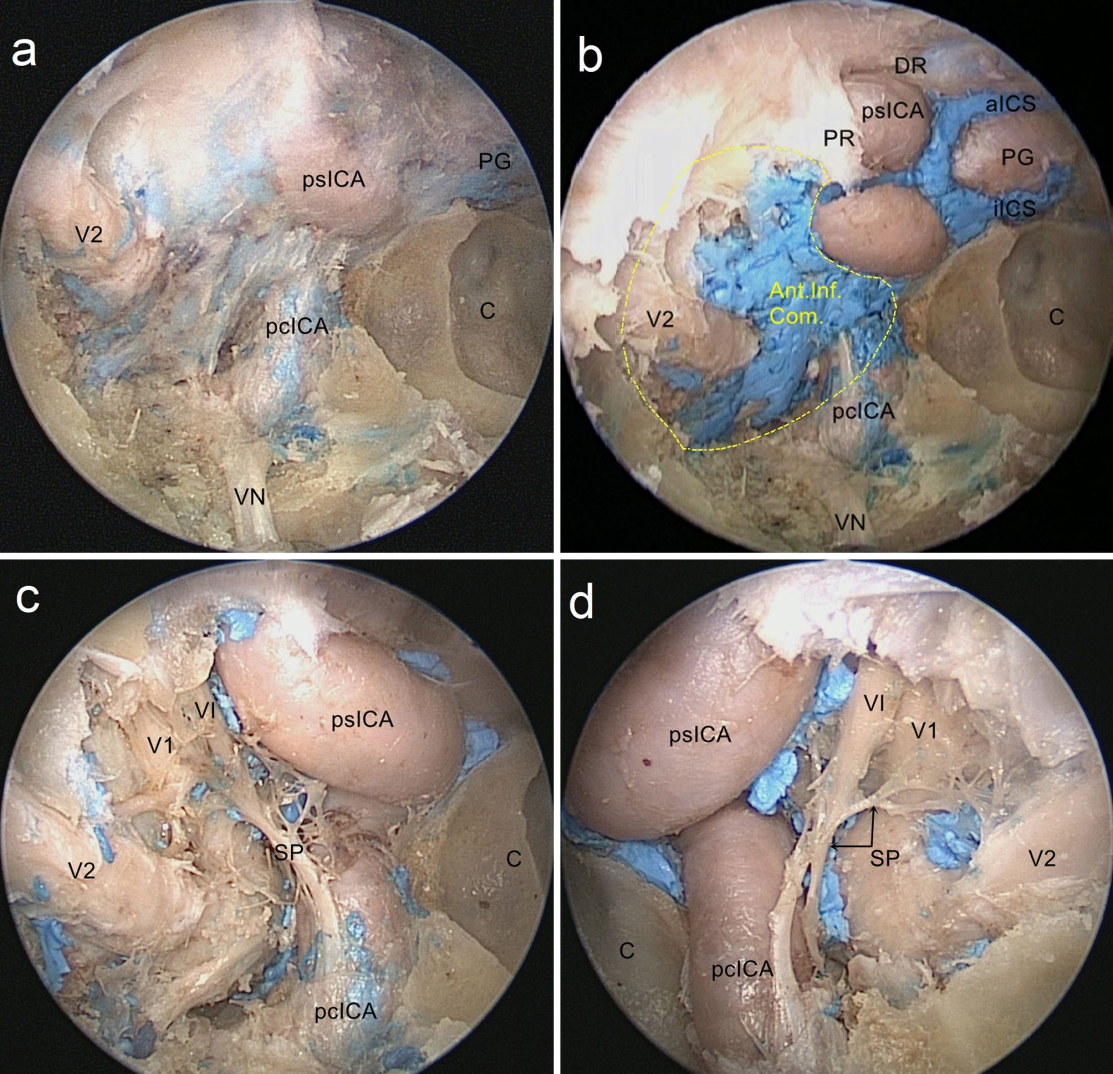
Endoscopic view of the anteroinferior compartment. (**a**) The nasal mucosa is bilaterally elevated from the vomer along the inferior wall of the sphenoid sinus up to identify the Vidian nerve. (**b**) The anteroinferior compartment of the CS is shaped like boat's bow (the yellow dotted line). (**c**) The sympathetic plexus is in this compartment around the ICA as it travels from the psICA to the ihsICA. (**d**) The sympathetic nerve fibers leave the internal carotid artery (ICA) and then join the abducens nerve and the first trigeminal division. *psICA* parasellar segment of the ICA, *pcICA* paraclival segment of the ICA, *PG* pituitary gland, *C* clivus, *DR* distal ring, *PR* proximal ring, *VN* Vidian nerve, *aICS* anterior intercavernous sinus, *iICS* inferior intercavernous sinus, *V1* ophthalmic branch of the trigeminal nerve, *V2* maxillary branch of the trigeminal nerve, *VI* abducens nerve, *SP* sympathetic plexus, *Ant.Inf.Com.* anteroinferior compartment of the CS.

The medial wall of the CS was removed. The anteroinferior compartment which was superior to the bottom of CS and inferior to the proximal dural ring of the CS venous space resembled a boat's bow. It was anteriorly limited by the CS anteroinferior wall, and posteriorly by the anterior surface of the anterior vertical psICA (Fig. 6b). The first nerve we encountered in the anteroinferior compartment around the ICA was the sympathetic plexus; it extended from the pcICA to the ihsICA. The sympathetic nerve was located medial to the abducens nerve with an oblique trajectory. It ran from the surface of the ICA and joined the ophthalmic nerve, with a more horizontal trajectory in this segment (Fig. 6c,d). We identified that the nasal mucosa was bilaterally elevated from the vomer along the inferior wall of the sphenoid sinus up to the vidian canal. The vidian nerve is formed by the union of the greater superficial petrosal nerve and the deep petrosal nerve and is a useful landmark to access the lacerum segment of the ICA (Figs. 2, 6a).

### Stage 4: Exposure of posterosuperior and lateral compartments of the CS venous spaces

The oculomotor, trochlear, ophthalmic (first trigeminal division), and maxillary (second trigeminal division) nerves are embedded in the reticularis membrane forming the lateral compartment of the CS; the abducens nerve, the intracavernous carotid artery, and the surrounding sympathetic plexus have a true intracavernous course. The oculomotor nerve, trochlear nerve, the first division of the trigeminal nerve, and abducens nerve formed a nerve bundle at the superior orbital fissure. Gentle medial mobilization of the ICA genu is required to gain access to the lateral compartment of the CS venous. The lateral compartment is lateral to the anterior vertical psICA and medial to the CS lateral wall. It includes ILT and the 4 triangles (the supratrochlear, infratrochlear, anteromedial, and anterolateral triangles).

**Figure 7:**
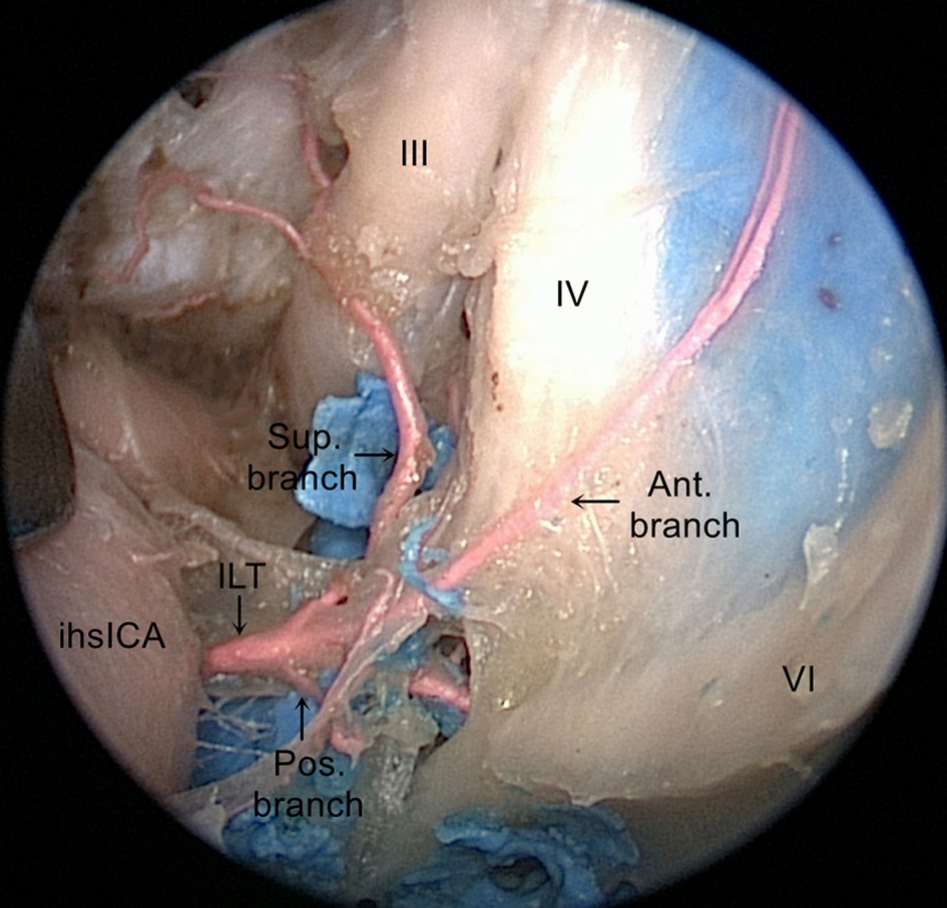
Endoscopic view of the ILT and its branches, the ILT originates from the lateral aspect of the ihsICA, passes above the abducens nerve, and is composed of three branches (superior, anterior and posterior). *ihsICA* inferior horizontal segments of the ICA, *III* oculomotor nerve, *IV* trochlear nerve, *VI* abducens nerve, *ILT* inferolateral trunk, *Sup.branch* superior branch of the ILT, *Ant.branch* anterior branch of the ILT, *Pos.branch* posterior branch of the ILT.

The ILT, also known as the inferior artery of the cavernous sinus, arises laterally from the ihsICA, and runs above the abducens nerve. The ILT supplies blood to the oculomotor, trochlear, abducens, and trigeminal cranial nerves, middle cranial base, cavernous courses, and middle skull base dura mater. The ILT comprises the superior, anterior, and posterior branches. The superior branch supplies blood to the CS roof and the oculomotor and trochlear nerves as they enter the cavernous sinus area. The anterior branch gives rise to a branch for the SOF and its contents and the posterior branch for the foramen ovale and its contents (Fig. 7).

**Figure 8:**
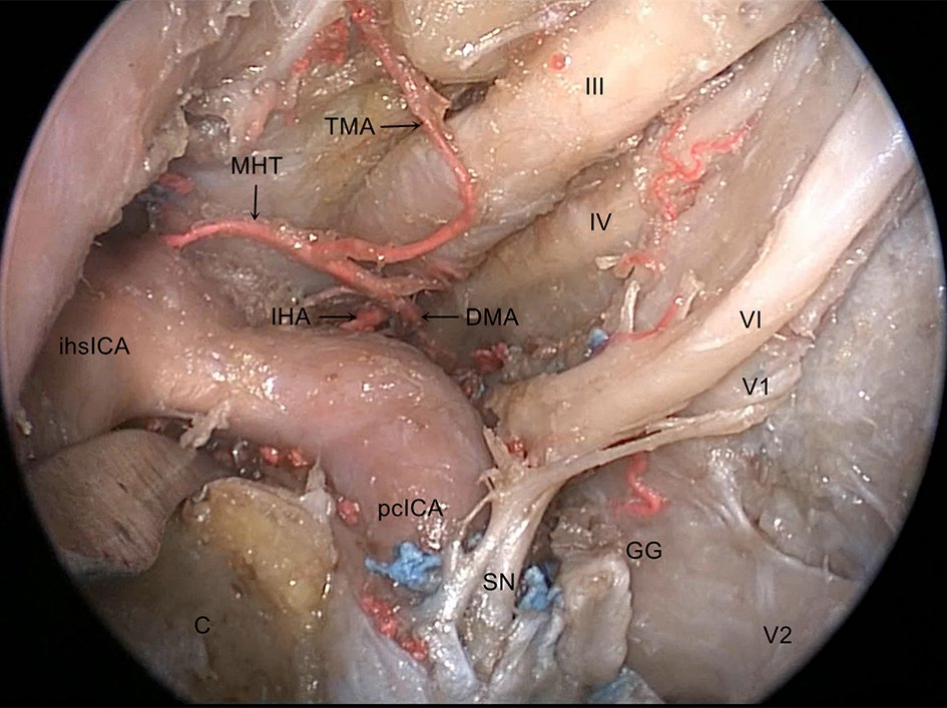
Endoscopic view of the MHT and three branches, the MHT arises from the posterior wall of the ihsICA and then divides into three branches: the inferior hypophyseal artery, dorsal meningeal artery and tentorial marginal artery. *pcICA* paraclival segment of the ICA, *ihsICA* inferior horizontal segments of the ICA, *C* clivus, *III* oculomotor nerve, *IV* trochlear nerve, *V1* ophthalmic branch of the trigeminal nerve, *V2* maxillary branch of the trigeminal nerve, *GG* gasserian ganglion, *VI* abducens nerve, *SN* sympathetic nerve, *MHT* meningohypophyseal trunk, *IHA* inferior hypophyseal artery, *DMA* dorsal meningeal artery, *TMA* tentorial marginal artery.

The posterosuperior compartment of the CS lies posterior to the anterior vertical psICA and anterior to the lateral clival dura; it continues medially with the medial compartment and laterally with the lateral compartment. It is related to the MHT and communicates with the opposite compartment through the basilar sinus. Through a gentle medial mobilization of the psICA, the MHT arises from the posterior wall of the ihsICA and then divides into three branches: the inferior hypophyseal artery, dorsal meningeal artery, and tentorial marginal artery. The inferior hypophyseal artery has a lateromedial trajectory toward the dura of the sellar floor, while the dorsal meningeal artery has a posterior and inferomedial trajectory towards the dura of the dorsum sella. These two arteries, along with the tentorial artery, either arise together from the MHT or as independent branches directly from the ICA. The tentorial marginal artery extends along the lateral wall of the CS towards the tentorium cerebelli (Fig. 8).

## Discussion

The CS is a valveless, dural-lined, venous plexus located near the center of the head, on either side of the sella, pituitary gland, and sphenoid sinus. The cavernous segment of the ICA lies medial to the CS and is surrounded by a periarterial sympathetic plexus. Courses of Cranial nerves III, IV, V1, and V2 run from top to bottom within the lateral dural walls of each CS. Cranial nerve VI lies immediately inferolateral to the ICA and is the only cranial nerve located within the venous sinusoids of the CS. The CS extends frontally from the superior orbital fissure and extends at the back of the area lateral to the dorsum sellae^20,21^.

The CS walls are defined differently in different studies. Campero et al. suggest that the external structure of each CS comprises of four walls of dura mater: the lateral, medial, superior (also called the roof of the CS), and the posterior walls^22^; Yasuda et al. consider a five-walled CS: lateral and medial walls, a roof, and posterior and anterior walls^23^; Chung BS et al. define the walls of the CS using sectioned images and three-dimensional (3D) models, where they believe that the CS was a hexahedron with six walls: lateral, medial, posterior, anterior, superior, inferior walls^24^. In this study, we proposed an endonasal classification of four walls for practical and surgical application: medial, anteroinferior, posterosuperior, and lateral walls. This may help in both preoperative and intraoperative identifications of CS walls and the corresponding venous spaces.

The classification of the CS venous spaces by Harris and Rhoton in 1976, is based on differentiated posterosuperior, anteroinferior, and medial venous spaces with the ICA^25^. Juan C Fernandez-Miranda et al.^26^ classify the four CS compartments based on their spatial orientation to the cavernous ICA: superior, posterior, inferior, and lateral compartments. Cavallo LM et al.^27^, report that the meningeal wall of the CS has three weak points for tumor invasion and extension: the venous plexus around the SOF, the loose texture of the medial wall around the pituitary body, and the dural pockets of the cranial nerves. The medial wall of the cavernous sinus is formed by a single, continuous, and thin layer of the dura, while the lateral and the superior walls are two-layered (meningeal and endosteal). The thin dura in the medial wall may be incomplete or absent. Most nonfunctioning pituitary adenomas extending into the cavernous sinus, are neither aggressive nor invasive, the lesions grow through the weak points of the walls of the cavernous sinus. While Ceylan et al.^28^ showed the integrity of the medial wall and its ultrastructural integrity with reverse transcription polymerase chain reaction (RT-PCR). Type IV collagen was considered to be an important factor in the progression of adenoma and invasion. The medial compartment has vital clinical utility because pituitary adenomas often invade the medial compartment of the CS through the venous connections by anteroinferior, posterior, and lateral extensions. Therefore, we classified the CS into four walls: medial, anteroinferior, posterosuperior, and lateral walls; and correspondingly the CS venous spaces into four compartments: medial, anteroinferior, posterosuperior, and lateral compartments.

Evidence of venous bleeding from the cavernous sinus is different. Carotid artery injury can occur when bone removal over the CS and along Meckel cave is being performed. Careful use of Kerrison rongeurs and drills is mandatory. Torquing or twisting motion with rongeurs should be avoided because such maneuvers can lead to shards of bone being driven into the vessel wall resulting in laceration or avulsion injuries^29^. Care should be exercised when working on the medial compartment of the CS venous space as follows:

1. During the dural opening of the upper and lower parts of the C-shaped medial compartment, the prominent anterior or inferior intercavernous sinuses pose limitations and may contribute to incomplete tumor resections (Figs. 3b and 6b). The bipolar forceps are used to reduce the risk of preoperative bleeding after an initial small dural incision is made^30^.
2. The superior hypophyseal artery is more likely to arise from the clinoidal segment of the ICA proximal to the DDR (Fig. 5c). It is remarkably difficult to avoid its accidental damage at the origin, especially with bipolar coagulation, while operating on the upper end of the C-shaped medial compartment^31^.
3. It is critical to identify the relationship between the lateral surface of the PG and the ihsICA before the surgery, to design the surgical procedure accordingly (Fig. 5). The indentation group may alter the course of the ICA. Vascular injury is one of the major concerns, especially due to the diverse relationship between the lateral surface of the PG and bilateral ihsICAs; this was found in 20% of the total specimens.

We speculated that distance measurements from the bilateral cavernous ICAs to the mid-line of the PG may benefit and support surgeons in extended approaches, and thus improve resection rates of lesions in this region. Thus, the distance from the bilateral cavernous ICAs to the mid-line of the PG was determined; these results are shown in Table 1. The difference between distances A (the distance from the medial border of the ssICA to the mid-line of the PG) and C (the distance from the medial border of the ihsICA to the mid-line of the PG) was not significant (5.70 ± 1.15 mm vs. 6.02 ± 1.73 mm); the distance from the medial border of the anterior vertical segment of cavernous ICA to the mid-line of the PG was10.37 ± 1.79 mm. If possible, vascularized reconstruction could be used (often the nasoseptal flap) to cover and protect the ICA when there is vascular exposure.

The anteroinferior compartment of the CS venous space resembled a boat's bow, and its surgery requires the removal of the bone that covers the anterior wall of the CS. Sympathetic fiber bundles travel along the surface of the carotid as it emerges from the foramen lacerum. Some of the bundles join the abducens nerve within the sinus and are ultimately distributed to the ophthalmic branch of the trigeminal nerve, which sends sympathetic fibers to the pupillodilator through the long ciliary nerves and the ciliary ganglion (Fig. 6c,d). The vidian nerve which consistently marks the level of the lacerum segment of the ICA in the petrous bone is one of the most important surgical landmarks to navigate the anteroinferior compartment^32^.

Our study showed that expanded endoscopic endonasal transsphenoidal approach provided excellent views of the medial and anteroinferior compartments of the CS venous spaces, with peculiar morphological characteristics. The medial compartment was C-shaped while the anteroinferior compartment of the CS resembled a boat's bow. These may help surgeons to accurately locate the neurovascular structures during endoscopic CS surgery. However, for the posterosuperior compartment of the CS venous spaces, which is frequently obliterated by the ICA, the endoscopic supraorbital extradural approach may offer better exposures^33^; the lateral compartment of the CS venous spaces is very narrow, thus, the transethmoid-pterygoid-sphenoidal endoscopic approach^5^ or endoscopic transorbital approach^34^ may be better for maneuverability. Several studies indicate that the posterior compartment may be an important venous compartment as well^26,35^. Especially recently, isolated posterior compartment involvement has also been reported in clinical series^36^. However, the posterior compartment has not been studied in this article.

The expanded endoscopic endonasal transsphenoidal approach should be performed only by experienced and well-trained neurosurgeons and otolaryngologists. This can be done by mastering the use of the endoscope in several endoscopic endonasal surgeries and endoscopic cadaver dissections. We believe that morphological characteristics like the one we found and those from other studies have potential clinical applicability in improving the safety of endoscopic skull base surgeries.

## Data Availability

The authors confirm that all data generated or analysed during this study are included in this published article and its supplementary materials.

## References

[1] Abuzayed B (2010). Expanded endoscopic endonasal approach to the clival region. J. Craniofac. Surg..

[2] Taniguchi M, Kohmura E (2012). Endoscopic endonasal removal of laterally Expanded clival chordoma using side-viewing scopes. Acta Neurochir. (Wien)..

[3] Cavallo LM (2007). The expanded endoscopic endonasal approach to the clivus and cranio-vertebral junction: Anatomical study. Childs Nerv. Syst..

[4] Kamat A (2015). Reconstructive challenges in the Expanded endoscopic transclival approach. J. Laryngol. Otol..

[5] Shin M (2017). Endoscopic transsphenoidal anterior petrosal approach for locally aggressive tumors involving the internal auditory canal, jugular fossa, and cavernous sinus. J. Neurosurg..

[6] de Notaris M (2009). Endoscopic endonasal transclival approach and retrosigmoid approach to the clival and petroclival regions. Neurosurgery.

[7] de Lara D (2014). Endonasal endoscopic approaches to the paramedian skull base. World Neurosurg..

[8] Pamias-Portalatin E (2018). Endoscope-assisted contralateral transmaxillary approach to the clivus and the hypoglossal canal: technical case report. J Neurosurg..

[9] Inoue A (2016). Role of denosumab in endoscopic endonasal treatment for juvenile clival giant cell tumor: A case report and review of the literature. World Neurosurg..

[10] Saito K, Toda M, Tomita T, Ogawa K, Yoshida K (2012). Surgical results of an endoscopic endonasal approach for clival chordomas. Acta Neurochir. (Wien)..

[11] Hong Jiang W (2009). Endoscopic resection of chordomas in different clival regions. Acta Otolaryngol..

[12] Fernandes Cabral DT (2018). Endoscopic endonasal transclival approach for resection of a pontine glioma: surgical planning, surgical anatomy, and technique. Oper. Neurosurg. (Hagerstown).

[13] Ciporen J, Lucke-Wold B, Dogan A, Cetas JS, Cameron WE (2016). Dual endoscopic endonasal transsphenoidal and precaruncular transorbital approaches for clipping of the cavernous carotid artery: A cadaveric simulation. J. Neurol. Surg. B Skull Base.

[14] Mendelson ZS, Patel AA, Eloy JA, Liu JK (2015). Endoscopic palliative decompression of the cavernous sinus in a rare case of a metastatic renal cell carcinoma to the clivus. Br. J. Neurosurg..

[15] Nakamura S, Kawamata T, Kobayashi T, Hori T (2010). Clival inflammation with cavernous sinus thrombophlebitis and orbital subperiosteal abscess–case report. Neurol. Med. Chir. (Tokyo).

[16] Yildirim AE, Divanlioglu D, Cetinalp NE, Ozhamam E, Belen AD (2014). Endoscopic endonasal treatment of a large clival giant cell tumor invading the cavernous sinus and temporal lobe. J. Craniofac. Surg..

[17] Ishii Y, Tahara S, Teramoto A, Morita A (2014). Endoscopic endonasal skull base surgery: Advantages, limitations, and our techniques to overcome cerebrospinal fluid leakage: Technical note. Neurol. Med. Chir. (Tokyo).

[18] Yang Y (2015). Morphological characteristics of the sphenoid sinus and endoscopic localization of the cavernous sinus. J. Craniofac. Surg..

[19] Alfieri A, Jho HD (2001). Endoscopic endonasal cavernous sinus surgery: An anatomic study. Neurosurgery.

[20] Yasuda A, Campero A, Martins C, Rhoton AL, Ribas GC (2004). The medial wall of the cavernous sinus: Microsurgical anatomy. Neurosurgery.

[21] Munawar K (2020). Cavernous sinus lesions. Clin. Imaging.

[22] Campero A, Campero AA, Martins C, Yasuda A, Rhoton AL (2010). Surgical anatomy of the dural walls of the cavernous sinus. J. Clin. Neurosci..

[23] Yasuda A (2005). Microsurgical anatomy and approaches to the cavernous sinus. Neurosurgery.

[24] Chung BS, Chung MS, Park JS (2015). Six walls of the cavernous sinus identified by sectioned images and three-dimensional models: Anatomic report. World Neurosurg..

[25] Harris FS, Rhoton AL (1976). Anatomy of the cavernous sinus. A microsurgical study. J. Neurosurg..

[26] Fernandez-Miranda JC (2018). Cavernous sinus compartments from the endoscopic endonasal approach: Anatomical considerations and surgical relevance to adenoma surgery. J. Neurosurg..

[27] Cavallo LM (2005). Endoscopic transnasal approach to the cavernous sinus versus transcranial route: Anatomic study. Neurosurgery.

[28] Ceylan S (2011). Microsurgical anatomy of membranous layers of the pituitary gland and the expression of extracellular matrix collagenous proteins. Acta Neurochir. (Wien).

[29] Lobo B, Zhang X, Barkhoudarian G, Griffiths CF, Kelly DF (2015). Endonasal endoscopic management of parasellar and cavernous sinus meningiomas. Neurosurg. Clin. N. Am..

[30] Chenin L, Toussaint P, Lefranc M, Havet E, Peltier J (2021). Microsurgical anatomy of the inferior intercavernous sinus. Surg. Radiol. Anat..

[31] Truong HQ (2018). Surgical anatomy of the superior hypophyseal artery and its relevance for endoscopic endonasal surgery. J. Neurosurg..

[32] Rhoton AL (2002). The cavernous sinus, the cavernous venous plexus, and the carotid collar. Neurosurgery.

[33] Komatsu F, Komatsu M, Inoue T, Tschabitscher M (2011). Endoscopic supraorbital extradural approach to the cavernous sinus: A cadaver study. J. Neurosurg..

[34] Caklili M, Emengen A, Cabuk B, Anik I, Ceylan S (2021). Endoscopic transorbital approach to the cavernous sinus lateral compartment (anatomical cadaver study). Turk. Neurosurg..

[35] Ceylan S, Anik I, Cabuk B, Caklili M, Anik Y (2019). Extension pathways of pituitary adenomas with cavernous sinus involvement and its surgical approaches. World Neurosurg..

[36] Yuanzhi X (2022). Intracranial breakthrough through cavernous sinus compartments: anatomic study and implications for pituitary adenoma surgery. Oper. Neurosurg..

